# Lymphoma Masquerading as an Ear Mass

**DOI:** 10.7759/cureus.14180

**Published:** 2021-03-29

**Authors:** Karen N Cuartas, Nathaniel R Wilson, Jaya Kala

**Affiliations:** 1 Internal Medicine, University of Texas Health Science Center at Houston, Houston, USA

**Keywords:** clinical case report, delayed diagnosis, diffuse large b cell lymphoma, ear neoplasm, incidental finding, non-hodgkin lymphoma

## Abstract

Due to the infrequently reported location, malignancies of the ear are usually misdiagnosed at the time of first presentation. To the best of our knowledge, there have been no reports in literature regarding diffuse large B-cell lymphoma (DLBCL) presenting as an ear mass, as was seen in our patient. We describe a case of a 38-year-old gentleman who presented with four months of worsening dyspnea on exertion and nonproductive cough. On exam there was a 4 cm x 5 cm erythematous, non-tender, and immobile mass on the right lower ear in the intertragic notch, sparing the lobe. CT of the neck and chest revealed prominent cervical lymph nodes and a diffusely spread circumferential soft tissue mediastinal mass involving the lungs, pleura, and pericardium. Malignancy was suspected, so the right ear mass was biopsied. Findings were consistent with DLBCL, germinal center B-type. This case provides a rare example of DLBCL presenting as an ear mass in a 38-year-old male with a chronic cough. We believe that prompt radiological evaluation of the chronic nonresolving cough may have helped in timely diagnosis of the malignancy, possibly halting the extensive infiltrative spread of disease, and thereby reducing the morbidity that the patient eventually suffered.

## Introduction

Diffuse large B-cell lymphoma (DLBCL) makes up around 30% of all cases of Non-Hodgkin Lymphoma, with roughly 150,000 new cases diagnosed globally each year [1]. Typically, DLBCL presents with a rapidly growing mediastinal mass with lymphadenopathy and systemic symptoms. However, masses may present anywhere in the body, including the head, neck, skin, and abdomen [1]. Due to the infrequently reported location, malignancies of the ear are usually misdiagnosed at the time of first presentation [2]. In this report, we describe the presentation of a patient with a right lower ear mass, which was later found to be the first extranodal manifestation leading to the diagnosis of primary mediastinal large B-cell lymphoma.

## Case presentation

A 38-year-old man with no past medical history presented to the ED for four months of progressively worsening dyspnea on exertion and nonproductive cough which increased at night. During the past month, he also noticed the gradual development of a nontender erythematous right ear mass, which he had not been aware of previously. He had lost 20 pounds of weight in the past eight months but did not report night sweats or cervical or axillary lymphadenopathy. There was no associated dysphagia, headaches, changes in vision or hearing, sputum production, or hemoptysis. Family history was significant for a sister diagnosed with cervical cancer at age 28.

He had seen his primary care physician at the onset of cough and dyspnea, who suggested over the counter cough suppressants for a presumed viral illness; no further laboratory or radiological investigation was done at the time of initial presentation. When his symptoms worsened, his primary care physician advised him to go to the ED for further evaluation.

**Figure 1:**
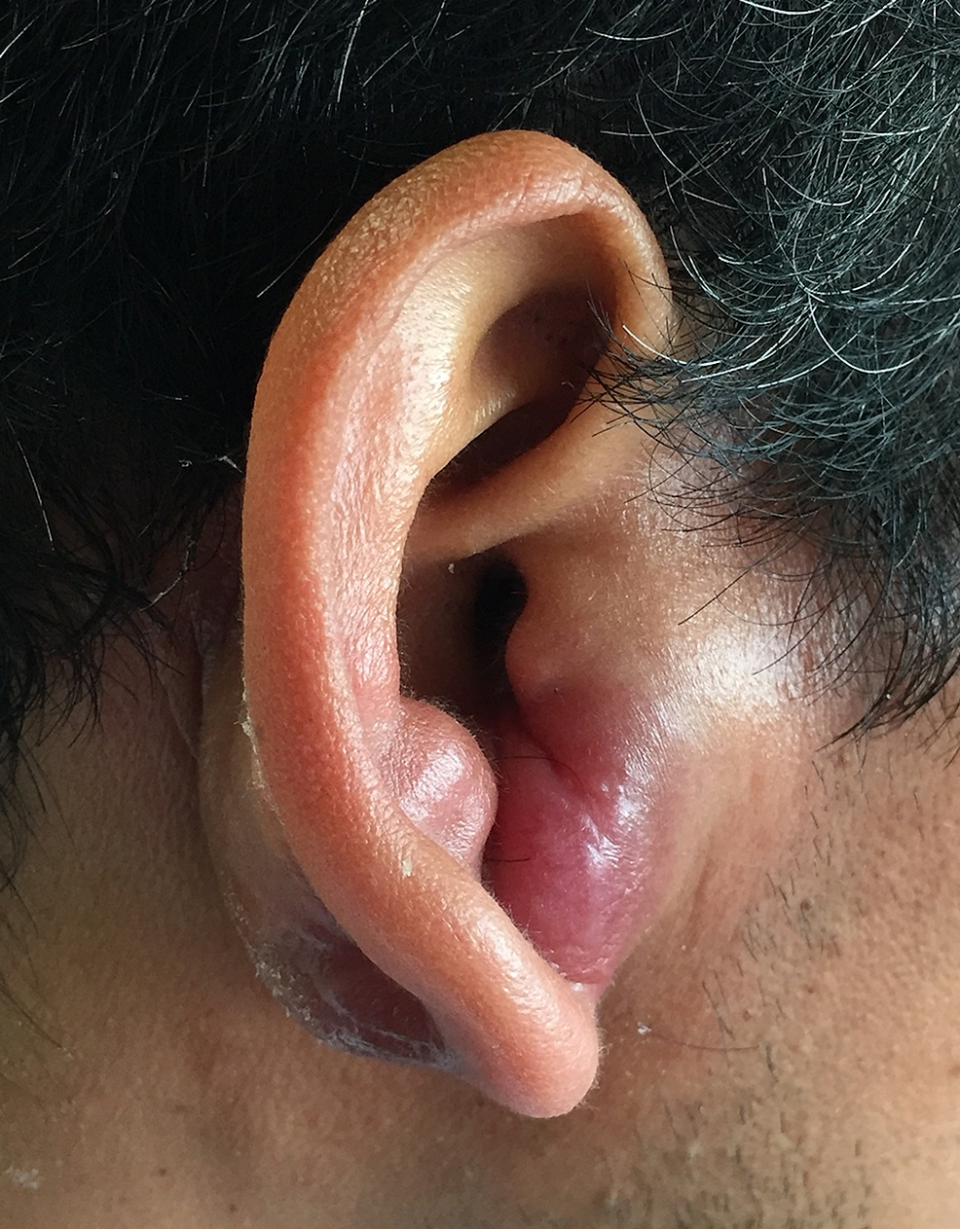
Right earlobe mass. Large, non-tender, immobile, 4 cm x 5 cm, erythematous mass on the right lower ear in the intertragic notch, sparing the lobe

His examination was significant for a heart rate of 110 beats per minute and decreased breath sounds in the right lower lung field. Of note, there was a nontender, immobile, 4 cm x 5 cm, erythematous mass on the right lower ear in the intertragic notch, sparing the lobe (Figure 1). No cervical lymphadenopathy was appreciated upon palpation.

**Figure 2:**
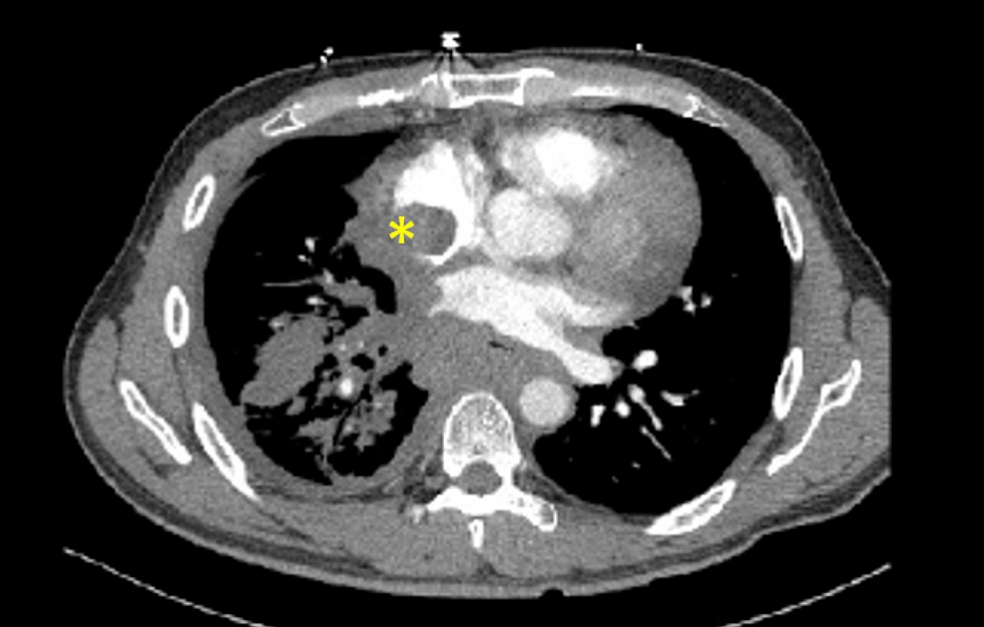
CT demonstrating large diffuse mediastinal mass. CT chest imaging demonstrating a diffusely spread circumferential soft tissue mediastinal mass involving the lungs, pleura, and pericardium. The mediastinal mass surrounds the esophagus, trachea, right pulmonary artery and vein, with extension into the bilateral atria (yellow asterisk).

CT of neck and chest revealed prominent cervical lymph nodes and a diffusely spread circumferential soft tissue mediastinal mass involving the lungs, pleura, and pericardium. The mediastinal density surrounded the esophagus, trachea, right pulmonary artery and vein, with extension into the bilateral atria (Figure 2). Further imaging with transthoracic echocardiography (TTE) revealed left ventricular ejection fraction of 55%-60% and confirmed infiltration of the mediastinal mass into the right and left atria. Cardiac MRI ruled out presence of intra-atrial thrombus. Abdominal MRI demonstrated extension of bulky disease below the diaphragm encasing the aorta, superior mesenteric artery, renal arteries, and vein.

The right ear mass was biopsied, revealing large cells which were positive for BCL-2, BCL-6, MUM1, CD20, CD19, CD22, CD10 on immunohistochemistry. Fluorescence in-situ hybridization returned negative for amplification or rearrangement of c-MYC, BCL-2, and BCL-6. Ki-67 proliferative index was 60%. These findings were consistent with DLBCL, germinal center B-type. The patient was diagnosed with Ann Arbor stage III primary mediastinal DLBCL with extranodal involvement of the right ear.

The patient was started on pharmacologic anticoagulation with low molecular weight heparin for venous thromboembolism prophylaxis, and admitted for initiation of inpatient chemotherapy with etoposide, prednisone, vincristine, cyclophosphamide, rituximab, and intrathecal methotrexate (DA-R-EPOCH). Doxorubicin was omitted for the first cycle of chemotherapy given the intra-atrial and myocardial involvement of tumor burden. However, his atrial tumor invasion receded with one cycle of chemotherapy and doxorubicin was added back for further cycles.

He completed treatment with DA-R-EPOCH for two cycles, followed by four cycles of rituximab, cyclophosphamide, doxorubicin, vincristine, prednisone (R-CHOP) with partial response. He is still being followed under active surveillance and has stable control of his disease now 47 months after initial diagnosis.

## Discussion

Malignant spread of lymphoma represents 15% of head and neck cancers, making it the third most common malignancy of the head and neck region [2]. Primary mediastinal large B-cell lymphoma is uncommon, comprising around 6%-10% of all DLBCL [3]. Germinal center B-cell variant typically has a better prognosis than nongerminal center type of DLBCL [4]. This type of DLBCL presents with bulky tumors involving the anterior mediastinum that rapidly progress causing local compressive symptoms. Therefore, prompt recognition is important to avoid associated morbidity and mortality.

Extranodal sites are seen in approximately 40% of patients with DLBCL [5]. These are bulky, polypoid masses with a smooth mucosal surface [6]. The most common sites of extranodal spread of disease are the gastrointestinal tract, skin, bones, head and neck, central nervous system, skeletal system, and testicles [7]. Nearly any location in the body can serve as a rare site for extranodal spread of lymphoma. Extranodal sites in the head and neck area include the nasopharynx, Waldeyer ring, oral cavity, larynx, and middle ear [6]. There have been reports of malignant lymphoma presenting as acute otitis media, mastoiditis, and infiltration of the external auditory canal [8-9]. Due to the infrequently reported location, malignancies of the ear are usually misdiagnosed at the time of first presentation. To the best of our knowledge, there have been no reports in literature of lymphoma presenting as an earlobe mass, as was seen in our patient.

In addition to extranodal spread of lymphoma, the differential diagnosis for an ear mass in the absence of systemic or respiratory symptoms, should also include other primary cutaneous T-cell lymphomas (i.e. mycosis fungoides or primary cutaneous CD30+ lymphoproliferative disorders), and primary cutaneous B-cell lymphomas (i.e. primary cutaneous marginal zone lymphoma, primary cutaneous follicle center lymphoma, primary cutaneous DLBCL, leg type) [10]. Our patient's progressive respiratory symptoms, however, suggested further workup with chest imaging which helped lead to the ultimate underlying diagnosis of primary mediastinal DLBCL with extranodal involvement of the ear.

There were certain barriers that may have contributed to the delay in diagnosis of DLBCL. Our patient was first seen at a large university medical center hospital. Upon discharge he was given instructions to follow-up outpatient at a community hospital that could enroll him in a financial assistance program. Because of a language barrier, our patient misunderstood the discharge instructions given to him at the large academic hospital and presented to the community hospital’s ED instead of scheduling an outpatient appointment.

This community hospital serves patients who often struggle accessing healthcare resources, transportation, and translation services. Many avoid coming to the hospital when symptoms first present due to fears of deportation and the cost of treatment. These barriers to care can potentially contribute to a later diagnosis, poor prognosis, and difficult follow up. At the time of his DLBCL diagnosis, our patient lived with and took care of his elderly father. His sister was diagnosed with metastatic cervical cancer only four months prior to his own cancer diagnosis. Because of this, he typically did not have family to accompany him to appointments. Social work helped our patient schedule outpatient appointments and arrange transportation. Despite these barriers and family hardships, he was able to successfully receive cancer-directed treatment.

## Conclusions

Diffuse large B-cell lymphoma can present with extranodal involvement in patients, including the ear. Furthermore, rapidly growing mediastinal tumors may cause local compressive symptoms, leading to implications for morbidity and mortality. Our patient’s constellation of progressive symptoms of dyspnea, cough, and weight loss highlight the importance of comprehensive history taking and thorough physical examination in developing a differential diagnosis that may include the dermatologic findings of the ear, which developed later. We believe that prompt radiological evaluation of the chronic nonresolving cough in our patient, may have helped in timely diagnosis of the malignancy. This, along with a higher clinical suspicion for malignancy and prompt evaluation of the ear mass, could have led to an earlier diagnosis in the outpatient setting, possibly halting the extensive lymphomatous infiltrative spread and thereby reducing the morbidity that the patient eventually suffered.
